# Supplementary material for: MetaRibo-Seq measures translation in microbiomes
Source: Nat Commun. 2020 Jun 29;11:3268. doi: 10.1038/s41467-020-17081-z (PMC7324362; doi:10.1038/s41467-020-17081-z)
Supplement: Supplementary file 10 — Supplementary Data 7 [file 41467_2020_17081_MOESM10_ESM.zip › File2/Confidence_High_Taxonomy/404591_out.krona.html]

Javascript must be enabled to view this page.

members
magnitude
magnitudeUnassigned
count
unassigned
taxon
rank

404591\_out

8

2
superkingdom
8

8
1224
phylum

class
1236
8

order
91347
8


SRS1055050\_contig\_number\_contig-100\_1550.1551
1
8
family
543

genus
547
1


SRS1055050\_contig\_number\_contig-100\_30.31
1
539813
species

genus
561
6


SRS017521\_contig\_number\_33167SRS049402\_contig\_number\_12174SRS076876\_contig\_number\_contig-100\_667.668SRS144362\_contig\_number\_23166SRS146888\_contig\_number\_14526SRS147022\_contig\_number\_14255
species
562
6
